# Is Following a Cancer-Protective Lifestyle Linked to Reduced Cancer Mortality Risk?

**DOI:** 10.3389/ijph.2023.1605610

**Published:** 2023-02-14

**Authors:** Flurina Suter, Nena Karavasiloglou, Julia Braun, Giulia Pestoni, Sabine Rohrmann

**Affiliations:** ^1^ Division of Chronic Disease Epidemiology, Epidemiology, Biostatistics and Prevention Institute (EBPI), University of Zurich, Zurich, Switzerland; ^2^ Divisions of Epidemiology and Biostatistics, Epidemiology, Biostatistics and Prevention Institute, University of Zurich, Zurich, Switzerland; ^3^ Nutrition Group, Health Department, Swiss Distance University of Applied Sciences, Zurich, Switzerland

**Keywords:** cancer prevention, spatial analysis, cancer mortality, global Moran’s *I*, WCRF/AICR recommendations

## Abstract

**Objectives:** This study investigates the association between a cancer protective lifestyle (defined based on the revised World Cancer Research Fund (WCRF) and the American Institute for Cancer Research (AICR) cancer prevention recommendations) and mortality in Switzerland.

**Methods:** Based on the cross-sectional, population-based National Nutrition Survey, menuCH (*n* = 2057), adherence to the WCRF/AICR recommendations was assessed *via* a score. Quasipoisson regression models were fitted to examine the association of adherence to the WCRF/AICR recommendations with mortality at the Swiss district-level. Spatial autocorrelation was tested with global Moran’s *I*. Integrated nested Laplace approximation models were fitted when significant spatial autocorrelation was detected.

**Results:** Participants with higher cancer prevention scores had a significant decrease in all-cause (relative risk 0.95; 95% confidence interval 0.92, 0.99), all-cancer (0.93; 0.89, 0.97), upper aero-digestive tract cancer (0.87; 0.78, 0.97), and prostate cancer (0.81; 0.68, 0.94) mortality, compared to those with lower scores.

**Conclusion:** The inverse association between adherence to the WCRF/AICR recommendations and mortality points out the potential of the lifestyle recommendations to decrease mortality and especially the burden of cancer in Switzerland.

## Introduction

In 2019, cancer was the leading cause of death among men and second leading cause of death among women in Switzerland (28.5% and 22.5% of all deaths, respectively) (1). The main risk factors that contribute to cancer mortality, such as smoking, physical inactivity, and unhealthy diets, are modifiable lifestyle factors (2). Of all cancer deaths, 30%–50% are assumed to be preventable through lifestyle modification (3). Hence, there is a global interest in primary cancer prevention targeting modifiable risk factors.

In 2018, the World Cancer Research Fund (WCRF) and the American Institute for Cancer Research (AICR) published a revised version of their 2007 WCRF/AICR cancer prevention recommendations, providing updated guidelines on an overall healthy and cancer-protective lifestyle at the individual level (4). The ten recommendations aimed on reducing the global burden of cancer (4). Several studies investigated the association between adherence to the WCRF/AICR cancer prevention recommendations represented by an index and cancer risk (5, 6) or cancer mortality (7, 8) and reported the assumed inverse relationship. A higher index indicates greater concordance with the 2018 WCRF/AICR cancer prevention recommendations and therefore, is assumed to be associated with a healthier lifestyle and a decreased cancer risk.

In 2016, Lohse et al. observed an inverse association between adherence to the WCRF/AICR cancer prevention recommendations and cancer mortality for men living in Switzerland using data of the years 1977–1979 and 1983–1992 (9). However, the study by Lohse et al. made use of rather old and crudely assessed data on diet and lifestyle (9). To overcome this limitation, we examined the association between adherence to the WCRF/AICR recommendations and mortality in Switzerland using the first National Nutrition Survey, menuCH, which assessed dietary intake and lifestyle factors in a representative sample of the Swiss population in greater detail than previous studies (10).

## Methods

The current study was based on three data sets: the menuCH survey (2014–2015, *n* = 2057), the Swiss mortality data (2015–2019), and the Swiss census data (2015–2019) provided by the Federal Statistical Office (FSO). The data sets were linked at the district-level. The structure of this article followed the STROBE-nut guidelines (11).

### Study Design and Participants of menuCH

In 2014/2015, the first National Nutrition Survey, the menuCH study (*n* = 2057), was conducted in ten centres across Switzerland. The survey was a cross-sectional population-based study with a target sample of 4,627,878 Swiss residents of 18–75 years of age. The study included one questionnaire (12) and two 24-hour dietary recalls (24HDR) of a representative sample of the Swiss population (10). The target sample was representative for five age categories (18–29, 30–39, 40–49, 50–64, 65–75 years old), both sexes (male, female), the three main Swiss language regions (CH-German, CH-French, and CH-Italian), and the twelve most populous cantons of the seven major regions of Switzerland. Further details on the study recruitment have been published elsewhere (13).

### Data Collection in the menuCH Survey

Data from the menuCH participants were obtained by a questionnaire assessing lifestyle and sociodemographic factors and by two 24HDR, as described in previous studies (13–15). Briefly, in the self-administered questionnaire the following information were collected: participants’ sex (male, female), age (afterwards divided into eleven categories: 18–24, 25–29, 30–34, 35–39, 40–44, 45–49, 50–54, 55–59, 60–64, 65–69, 70–75 years old), language region (CH-German, CH-French, CH-Italian), nationality (Swiss, Non-Swiss, Swiss binational), civil status (single, married, divorced, other), education level (primary, secondary, tertiary), smoking status (never, former, current), physical activity (low, moderate, high; based on the short-form International Physical Activity Questionnaire (IPAQ) definitions (16)), and postal code.

Anthropometric factors were measured during the first 24HDR by trained personnel following standardized procedures (17). Self-reported weight or height measurements were used for lactating and pregnant women and for participants, for whom a measurement was not possible. Subsequently, body mass index (BMI) was calculated and classified according to the World Health Organization as “underweight” (BMI <18.5 kg/m^2^), “normal weight” (18.5 kg/m^2^ ≤ BMI <25.0 kg/m^2^), “overweight” (25.0 kg/m^2^ ≤ BMI <30.0 kg/m^2^) or “obese” (BMI ≥30 kg/m^2^) (18).

Dietary data were collected during two non-consecutive 24HDR interviews. The interviews were performed by trained dietitians across all weekdays and seasons using the trilingual Swiss version (0.2014.02.27) of the software GloboDiet® (formerly EPIC-Soft®, International Agency for Research on Cancer (IARC), Lyon, France (19, 20); adapted by the Federal Food Safety and Veterinary Office, Bern, Switzerland). The first interview was held on-site in one of the ten study centres, whereas the second interview was conducted two to 6 weeks later *via* telephone. For quantifying the consumed amounts, the participants were provided a book with about 60 actual household measures and 119 series of five to six graduated portion-sized pictures (13, 21). After data collection, its quality was ensured by cleaning the data based on the IARC’s guidelines using an updated version of GloboDiet® (0.2015.09.28) (22). In the end, obtained ingredients, foods, and recipes were matched to the most suited item found in the Swiss Food Composition Database (23) using the tool FoodCASE (Premotec GmbH, Winterthur, Switzerland). Assessment of dietary supplement intake was not included in the 24HDR, but was collected *via* self-administered questionnaire.

### Swiss Mortality and Census Data

To be consistent with the menuCH study, we used Swiss mortality and census data of the age range 18–75 years.

All-cause, all-cancer, and cancer-specific mortality based on the documented definitive cause of death were examined. Based on the existing evidence on health behaviour factors and their link to cancer prevention (2), the following cancer types encoded with the 10th revision of the International Classification of Diseases (ICD-10) (24) were investigated: all-cancer (ICD-10: C00-C97, D32-D33, and D37-D48), colorectal cancer (ICD-10: C18-C20), upper aero-digestive tract (UADT) cancer (including tissues and organs of the respiratory tract, upper part of the digestive tract, and the upper oesophagus; ICD-10: C00-C15 and C32), stomach cancer (ICD-10: C16), liver cancer (ICD-10: C22), pancreatic cancer (ICD-10: C25), breast cancer (only women; ICD-10: C50), and prostate cancer (only men; ICD-10: C61).

The mortality data provided by the FSO were linked to the dietary data by the place of residence of the participants using postal code information. Based on the indirect method, which uses the Swiss population as reference population, standardized mortality ratios (SMR) were calculated at the district-level. The SMR were standardized for sex, age, and year of death.

### 2018 WCRF/AICR Cancer Prevention Recommendations Score

In 2018, the WCRF and AICR published ten recommendations on cancer prevention (4), of which our study used the following seven to build an index: maintain a healthy weight, be physically active, eat a plant-based diet, and limit the consumption of fast-food, red and processed meat, sugar-sweetened drinks, and alcohol. In an additional sensitivity analysis, the recommendation on dietary supplement use was included. Our study excluded the recommendation on breastfeeding and the one pertinent to people with a previous cancer diagnosis, since the menuCH study did not provide enough information to determine the partial score of these two recommendations. To make studies more comparable and to consider interdependent effects of risk factors on cancer, Shams-White et al. (3, 25) defined an index reflecting adherence to the recommendations, to which each recommendation contributes equally. The WCRF/AICR score construction in menuCH was based on the index by Shams-White et al. (3, 25) and details were provided in a previous project (Karavasiloglou N et al., in review). Briefly, each of the seven included recommendations contributed equally to the final score. For each recommendation either 0, 0.5, or 1 point were assigned. Two equally weighted sub-recommendations were included for the healthy weight (based on BMI category and waist circumference) and the plant-based diet (based on fruits and vegetables intake and total fiber intake) recommendation each. Thus, for each of the latter two recommendations a partial score of 0.25 and 0.75 was possible, too (3, 25).

The physical activity recommendation was assessed using the short-form IPAQ. Dietary components were investigated as consumed average amount in grams per day (mean of the two 24HDR interviews). For the fast-food component, an adapted NOVA classification system was applied to categorize foods as ultra-processed or non-ultra-processed (Karavasiloglou N et al., in review). The NOVA classification was adapted to the cancer prevention recommendation score to ensure no double penalization for red and processed meat consumption and intake of sugar-sweetened drinks. Closer adherence to the WCRF/AICR cancer prevention recommendations lead to a higher score, indicating a healthier lifestyle. In the analyses, the score was used as a continuous variable, ranging from zero to seven points, and as a categorical variable with the following predefined three categories: low adherence (0-<3 points), moderate adherence (3-<5 points), and high adherence (5–7 points).

### Statistical Analyses

The menuCH participants’ characteristics and the SMR at the district-level were analysed descriptively.

To investigate the association between the cancer prevention score and all-cause and cancer mortality, Quasipoisson regression models, which are a generalized version of Poisson models, were fitted at the individual level. The outcome variable modeled by a Quasipoisson regression model is a count variable, that shows under- or overdispersion, i.e., the mean and the variance of the outcome data are not equal and therefore, the variance will be estimated by an additional parameter, the dispersion parameter. As the menuCH survey is not a longitudinal study and therefore lacks a mortality follow-up, an additional data set, the Swiss mortality data from 2015 until 2019, was used to determine the outcome variable. The Swiss mortality data were linked to the menuCH data geographically at the district level. The outcome variable was the observed number of deaths documented between 2015 and 2019 in the menuCH participant’s sex, age, and district group. The total number of residents in the corresponding sex, age, and district group was added as an offset term. The cancer prevention score was used as explanatory variable (either as continuous or categorical variable). The models were further adjusted for sex, age, smoking status, education level, language region, nationality, civil status, and mean daily energy intake (in kilocalories).

A sensitivity analysis excluding the physical activity recommendation due to a high percentage of missing observations was conducted. Furthermore, a second sensitivity analysis was performed, which included the seven previous recommendations and additionally the recommendation on dietary supplement use as a binary score (0 points if self-medicated intake; 1 point if prescribed by a doctor or no supplement intake).

Districts were specified as neighbouring based on a first order neighbourhood structure with rook contiguity. Furthermore, each neighbour district received a weight according to the inverse number of neighbours of the corresponding district. The global Moran’s *I* statistic was used to investigate the existence and degree of spatial autocorrelation (26). A one-sided *p*-value based on the Z-score (27) and a one-sided *p*-value based on 1000 Monte Carlo (MC) simulations were calculated to check the evidence for a significant, non-random spatial pattern of the residuals aggregated at the district-level. Local Moran’s *I* were calculated and tested for significance based on a permutation test (*n* = 1000). The lower limit of the *p*-value is given by the number of simulations (28) and therefore, no correction for multiple testing was applied. Local indicators of spatial autocorrelation (LISA) cluster maps were used to present the local Moran’s *I* values. “High-High” representing districts, which had a higher residual mean than the overall residual mean and a lagged value, which was higher than the overall mean lagged value. “Low-Low” indicating districts, for which both values were lower than the corresponding average value. “High-Low” representing districts, which had a higher residual mean than the overall mean and a lagged value, which was lower than the overall mean lagged value. “Low-High” representing districts, for which the opposite was the case. Districts, which were not part of the menuCH study, and thus no data were available, are coloured in white.

An integrated nested Laplace approximation (INLA) model was fitted when evidence for spatial autocorrelation was observed. A *Besag-York-Mollié* model was chosen, which consists of a structured spatial component and an unstructured spatial component (29). For both components, the default prior distribution (LogGamma with shape = 1 and rate = 0.00005) was chosen (29). For each data set the results were pooled by taking the average of the estimates.

The menuCH participants’ data were weighted for sex, age, major living region in Switzerland, marital status, household size, and nationality. Variables on dietary factors were additionally weighted for weekday and season of the 24HDR interview day (30).

Some participants had missing values for the physical activity level (*n* = 524), the education level (*n* = 3), and smoking category (*n* = 4). Hence, to include all 2057 participants in the analyses, we conducted multivariate imputation by chained equations (MICE, m = 25). With each imputed data set the analyses were run separately. Afterwards, the results were pooled.

Analyses were conducted in GeoDa (version 1.14.0) and in the software R (version 4.1.0, R Foundation for Statistical Computing, Vienna, Austria (31)). The statistical significance level was set to 0.05 for all analyses.

## Results

In comparison to the overall study population, several differences in the menuCH participants’ characteristics were observed across the adherence categories (Table 1). Participants in the low adherence group were more likely to be 30 years of age or older, live in a CH-German language region, be of Swiss nationality only, have completed a secondary education, be current smokers, and have a higher daily energy intake. In contrast, participants with high adherence were more likely to be female, 18–29 years of age, live in the CH-French language region, be Swiss binational, be single, have completed a tertiary education, be never-smokers, and have a lower daily energy intake.

**TABLE 1:** Characteristics of menuCH participants (*n* = 2057, unweighted count) stratified by the categories of adherence to the cancer prevention recommendations^a^ ^,^ ^b^. menuCH. Switzerland. 2014–2015.

Variables	Overall (*n* = 2057)	Low adherence^c^ (*n* = 227)	Moderate adherence^c^ (*n* = 900)	High adherence^c^ (*n* = 379)	NA (*n* = 551)
%	100	11.1	45.5	17.9	25.5
Women (%)	50.2	34.1	43.3	67.5	57.3
Age group (%)					
18–29 years old	18.8	13.0	17.7	23.2	20.2
30–44 years old	29.9	31.3	30.9	26.3	29.9
45–59 years old	29.8	32.6	30.7	28.6	27.8
60–75 years old	21.6	23.1	20.7	21.9	22.1
Language region^d^ (%)					
German	69.2	73.3	70.2	67.7	66.9
French	25.2	21.6	24.1	27.3	27.3
Italian	5.6	5.1	5.7	5.0	5.8
Nation group (%)					
Swiss only	61.4	67.4	59.4	60.8	62.9
Non-Swiss	24.8	21.6	26.5	24.8	23.1
Swiss binational	13.8	10.9	14.1	14.4	14.0
Civil status (%)					
Single	31.1	29.0	30.8	35.0	30.0
Married	52.2	53.0	54.0	48.0	51.7
Divorced	12.1	12.7	11.5	10.7	13.9
Other	4.4	5.3	3.7	6.3	3.9
NA	0.1	0.0	0.0	0.0	0.6
Education level (%)					
Primary	4.7	2.8	5.4	3.4	5.2
Secondary	42.6	48.4	39.4	36.1	50.3
Tertiary	52.6	48.8	55.3	60.5	44.0
NA	0.1	0.0	0.0	0.0	0.6
Smoking (%)					
Never	42.9	35.0	41.4	52.2	42.4
Former	33.6	34.1	32.9	34.0	34.5
Current	23.3	30.9	25.7	13.9	22.2
NA	0.2	0.0	0.0	0.0	0.8
Daily energy intake [kcal]	2130 (1711, 2600)	2413 (1940, 2791)	2163 (1758, 2623)	1930 (1580, 2354)	2042 (1637, 2597)

^a^ The cancer prevention recommendation score included the following 7 recommendations: healthy weight, physical activity, plant-based diet, limited consumption of fast-food, red and processed meat, sugar sweetened drinks, and alcohol. World Cancer Research Fund/American Institute for Cancer Research cancer prevention score categories: category 1: low adherence (0-<3 points); category 2: moderate adherence (3-<5 points); category 3: high adherence (5-7 points).

^b^ Categorical variables are expressed as weighted percentage (%). Continuous variables are expressed as weighted median and weighted interquartile range. The weighted results are weighted, as stated in the menuCH weighting strategy (30), for sex, age, major living region in Switzerland, marital status, household size, and nationality. Daily energy intake [kcal] was further weighted for weekday and season of the recall day.

^c^ World Cancer Research Fund/American Institute for Cancer Research cancer prevention score categories: low adherence corresponds to a score of 0-<3 points; moderate adherence corresponds to a score of 3-<5 points; high adherence corresponds to a score of 5-7 points.

^d^ German language region: cantons *Aargau*, *Basel City*, *Basel Country*, *Berne*, *Lucerne*, *Zurich*, and *St. Gallen*. French language region: cantons *Jura*, *Neuchâtel*, *Vaud*, and *Geneva*. Italian language region: canton *Ticino*.

NA, missing values.

**FIGURE 1:**
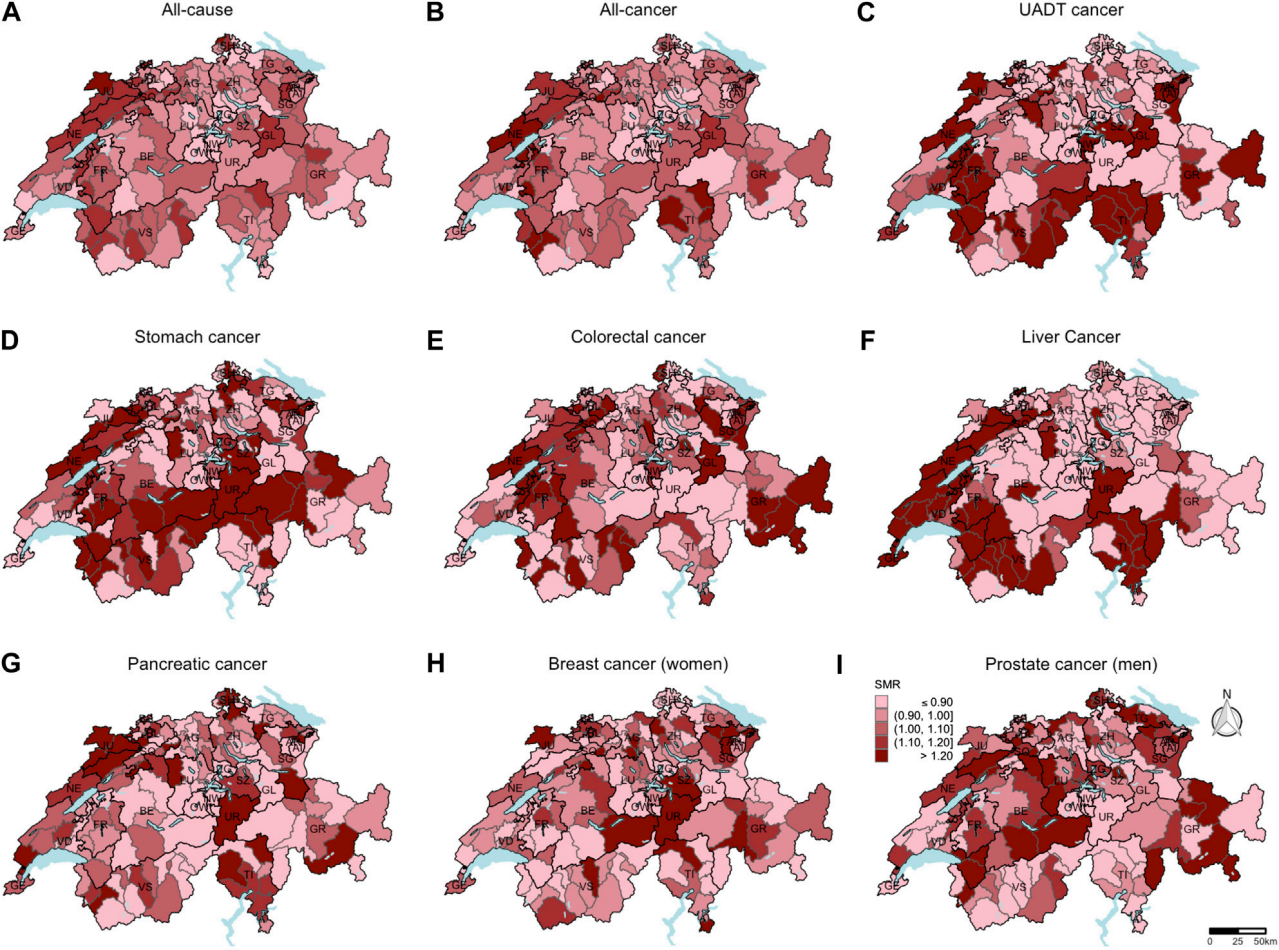
Standardized mortality ratios at the district-level (unweighted data number of districts = 143). Based on the indirect method using the Swiss population as reference population, mortality ratios standardized for sex, age, and year of death were computed. Breast cancer standardized mortality ratios **(H)** were calculated only for women. Prostate cancer standardized mortality ratios **(I)** were calculated only for men. For all other causes of death **(A–G)**, the data of both sexes were included to calculate the standardized mortality ratios. Swiss mortality and Swiss census data. Switzerland. 2015–2019.

From 2015 until 2019, 106,140 all-cause deaths, including 46,220 all-cancer, 4022 colorectal cancer, 2923 UADT cancer, 1525 stomach cancer, 2248 liver cancer, 3841 pancreatic cancer, 3818 breast cancer (among women), and 1913 prostate cancer deaths (among men) were reported. In Figure 1, the SMR for all-cause, all-cancer, and cancer-specific mortality are shown at the district-level. Higher all-cause and all-cancer SMR were mainly observed in the western region and lower SMR mainly in the central region of Switzerland. A clear distinction between the CH-German regions with low SMR and the CH-French and CH-Italian regions with high SMR was seen for liver cancer. For most of the individual cancer sites, no clear pattern was detectable.

Regarding the Quasipoisson regression models, the complete case analyses and the analyses based on imputed data revealed similar results, hence only the latter results are presented and used for subsequent analyses. When using the score as continuous variable, no statistically significant rate ratio was observed for any of the mortality outcomes (Table 2).

**TABLE 2:** Association of cancer prevention recommendation score^a^ with all-cause and cancer mortality (*n* = 2057, unweighted count) (rate ratios and 95% confidence intervals)^b^. menuCH. Switzerland. 2014–2015.

Mortality^d^	WCRF/AICR cancer prevention recommendations score				
	Continuous		Categorical^c^		
	Per 1-point increment RR (95% CI)		Low adherence (ref.) RR (95% CI)	Moderate adherence RR (95% CI)	High adherence RR (95% CI)
All-cause^e^ ^,^ ^f^	1.00 (0.99, 1.01)		1.00 -	0.99 (0.96, 1.02)	0.95 (0.92, 0.99)
All-cancer^e^ ^,^ ^f^	0.99 (0.98, 1.00)		1.00 -	0.99 (0.95, 1.02)	0.93 (0.89, 0.97)
UADT cancer^e^ ^,^ ^f^	0.99 (0.96, 1.01)		1.00 -	0.93 (0.85, 1.01)	0.87 (0.78, 0.97)
Stomach cancer^e^ ^,^ ^f^	0.98 (0.94, 1.02)		1.00 -	0.93 (0.81, 1.05)	0.85 (0.70, 1.00)
Colorectal cancer^e^ ^,^ ^f^	0.99 (0.96, 1.01)		1.00 -	1.08 (1.00, 1.15)	0.95 (0.87, 1.04)
Liver cancer^e^ ^,^ ^f^	0.99 (0.96, 1.02)		1.00 -	1.04 (0.95, 1.13)	0.92 (0.82, 1.03)
Pancreatic cancer^e^ ^,^ ^f^	1.01 (0.99, 1.03)		1.00 -	1.03 (0.96, 1.10)	1.00 (0.91, 1.08)
Breast cancer^e^ ^,^ ^g^	0.98 (0.96, 1.00)		1.00 -	1.04 (0.96, 1.13)	0.97 (0.88, 1.06)
Prostate cancer^e^ ^,^ ^h^	0.98 (0.95, 1.02)		1.00 -	0.86 (0.77, 0.96)	0.81 (0.68, 0.94)

^a^ The cancer prevention recommendation score included the following seven recommendations: healthy weight, physical activity, plant-based diet, limited consumption of fast-food, red and processed meat, sugar sweetened drinks, and alcohol.

^b^ The menuCH participants’ data were weighted, as stated in the menuCH weighting strategy (30), for sex, age, major living region in Switzerland, marital status, household size, nationality, weekday, and season of the recall day.

^c^ World Cancer Research Fund/American Institute for Cancer Research cancer prevention score categories: low adherence corresponds to a score of 0-<3 points; moderate adherence corresponds to a score of 3-<5 points; high adherence corresponds to a score of 5-7 points.

^d^ From 2015 until 2019, 106,140 all-cause deaths, including 46,220 all-cancer, 4022 colorectal cancer, 2923 UADT cancer, 1525 stomach cancer, 2248 liver cancer, 3841 pancreatic cancer, 3818 breast cancer (among women), and 1913 prostate cancer deaths (among men) were reported.

^e^ A Quasipoisson regression model was fitted.

^f^ The analyses included data of both sexes and were further adjusted for sex, age, smoking category, education level, language region, nationality, civil status, and mean daily energy intake.

^g^ The analysis included data of only women and was further adjusted for age, smoking category, education level, language region, nationality, civil status, and mean daily energy intake.

^h^ The analysis included data of only men and was further adjusted for age, smoking category, education level, language region, nationality, civil status, and mean daily energy intake.

UADT, upper aero-digestive tract; RR, rate ratio; CI, confidence interval; WCRF, World Cancer Research Fund; AICR, American Institute for Cancer Research.

In the regression models using the score as categorical variable, there was evidence among participants to have a 5% decrease in all-cause (RR = 0.95, 95% CI: 0.92, 0.99), a 7% decrease in all-cancer (RR = 0.93, 95% CI: 0.89, 0.97), a 13% decrease in UADT cancer (RR = 0.87, 95% CI: 0.78, 0.97), and a 19% decrease in prostate cancer (RR = 0.81, 95% CI: 0.68, 0.94) mortality when comparing the high adherence with low adherence group. For prostate mortality, even participants with moderate adherence had a reduced mortality by 14% compared to the low adherence group (RR = 0.86, 95% CI: 0.77, 0.96). Evidence for an increase in colorectal cancer mortality by 8% was observed in the moderate compared to the low adherence group (RR = 1.08, 95% CI: 1.00, 1.15). Apart from the latter result, there was an overall tendency for a decrease in mortality in the moderate adherence group and an even stronger decrease in mortality in the high adherence group compared to the low adherence group.

The results of the sensitivity analyses can be found in Supplementary Tables S1, S2. Generally, sensitivity analyses showed an attenuation of the statistical significance of the results.

Supplementary Tables S3, S4 show the results of the global Moran’s *I* statistic based on the regression models including the score as continuous and as categorical variable, respectively. Both models revealed similar results.

**FIGURE 2:**
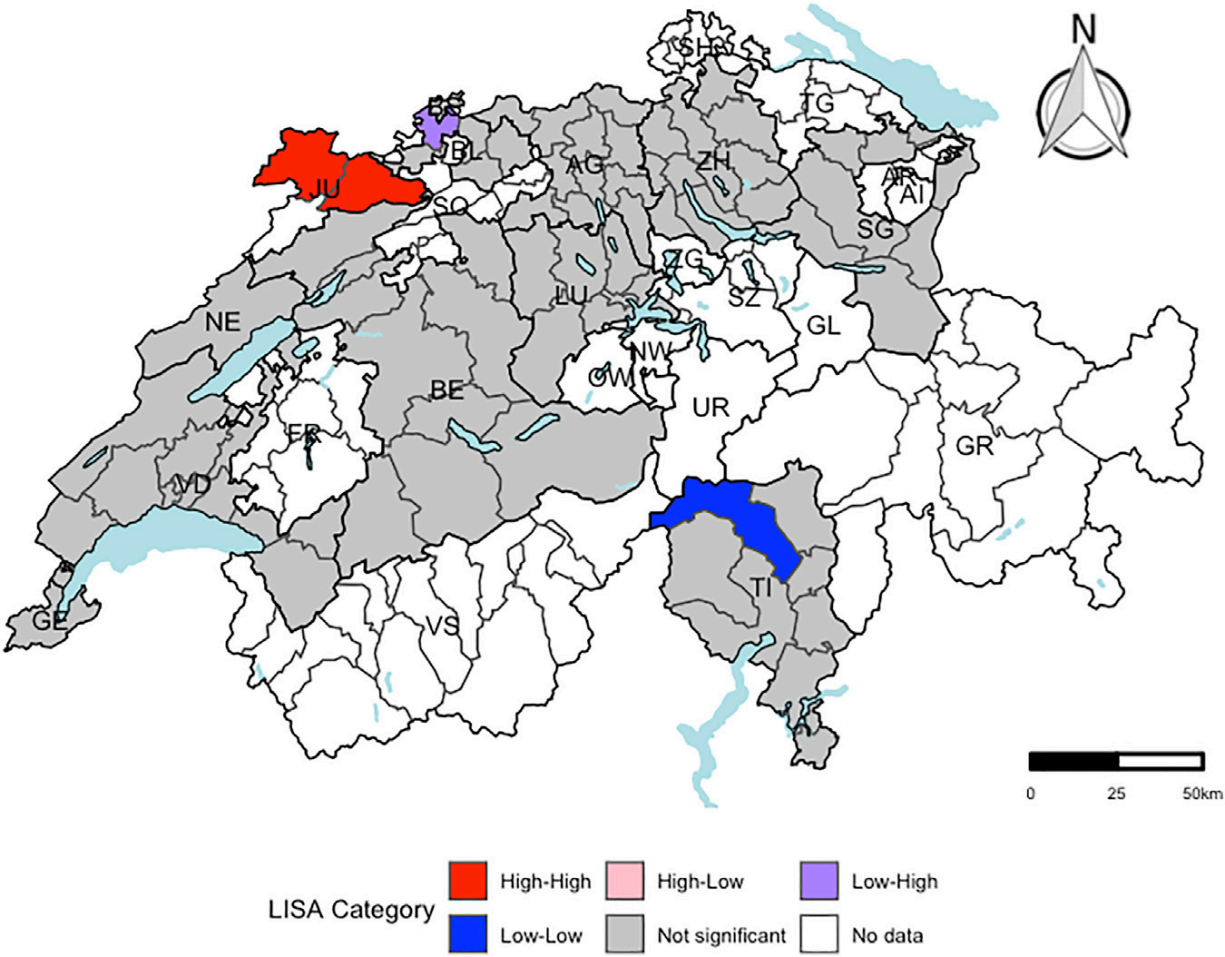
Visualization of local Moran’s *I* for liver cancer mortality using a local indicators of spatial autocorrelation cluster map at the district-level (respectively. *n* = 143). “High-High” representing districts, which had a higher residual mean than the overall residual mean and a lagged value, which was higher than the overall mean lagged value. “Low-Low” indicating districts, for which both values were lower than the corresponding average value. “High-Low” representing districts, which had a higher residual mean than the overall mean and a lagged value, which was lower than the overall mean lagged value. “Low-High” representing districts, for which the opposite was the case. Districts, which were not part of the menuCH study, are coloured in white. A significance level of 0.05 with no correction for multiple testing was applied. Independent of including the score as categorical or continuous outcome variable in the regression model, the same four Swiss districts revealed significant evidence for spatial outliers or spatial clusters. menuCH and Swiss mortality and Swiss census data. Switzerland. 2014–2015 and 2015–2019.

Only the residuals of the regression model for liver cancer mortality revealed evidence for spatial autocorrelation at the district-level (expected global Moran’s *I*: −0.014; observed global Moran’s *I*: 0.143 (categorical score) and 0.152 (continuous score), respectively). In Figure 2, the districts with a significant local Moran’s *I* statistic for liver cancer mortality are visualised in a LISA cluster map, providing more detailed information on the statistically significant spatial pattern indicated by the global Moran’s *I* statistic. Independent of including the score as categorical or continuous variable in the regression model, four districts revealed significant evidence for spatial outliers or spatial clusters, indicating to be the core of a spatial pattern.

INLA models were fitted for liver cancer mortality (Table 3). The fixed effects of the INLA model were similar as the estimates of the Quasipoisson model for both the continuous and the categorical model. There was still no evidence for an association between the score and liver cancer mortality. The structured spatial component of the INLA model did not reveal evidence for any of the Swiss districts to have increased or decreased liver cancer mortality.

**TABLE 3:** Integrated nested Laplace approximation model for association of cancer prevention recommendation score^a^ with sex-, age-, and district-specific liver cancer mortality: fixed effects (*n* = 2057, unweighted count) (rate ratios, 95% credible intervals)^b^. menuCH. Switzerland. 2014–2015.

Mortality^d^	WCRF/AICR cancer prevention recommendations score				
	Continuous		Categorical^c^		
	Per 1-point increment RR (95% CI)		Low adherence (ref.) RR (95% CI)	Moderate adherence RR (95% CI)	High adherence RR (95% CI)
Liver cancer^e^ ^,^ ^f^	0.98 (0.95, 1.01)		1.00 -	1.01 (0.92, 1.11)	0.89 (0.80, 1.00)

^a^ The cancer prevention recommendation score included the following seven recommendations: healthy weight, physical activity, plant-based diet, limited consumption of fast-food, red and processed meat, sugar sweetened drinks, and alcohol.

^b^ The menuCH participants’ data were weighted, as stated in the menuCH weighting strategy (30), for sex, age, major living region in Switzerland, marital status, household size, nationality, weekday, and season of the recall day.

^c^ World Cancer Research Fund/American Institute for Cancer Research cancer prevention score categories: low adherence corresponds to a score of 0-<3 points; moderate adherence corresponds to a score of 3-<5 points; high adherence corresponds to a score of 5-7 points.

^d^ From 2015 until 2019, 2248 liver cancer were reported.

^e^ An integrated nested Laplace approximation (INLA) model using the *poisson* family was fitted.

^f^ The analysis included data of both sexes and were further adjusted for sex, age, smoking category, education level, language region, nationality, civil status, and mean energy intake per day in kilocalories.

INLA, Integrated nested Laplace approximation; RR, rate ratio; CI, credible interval; WCRF, World Cancer Research Fund; AICR, American Institute for Cancer Research.

## Discussion

In this study, the association of the cancer prevention score with all-cause and cancer mortality in Switzerland was explored. Quasipoisson regression models revealed evidence for a decrease in all-cancer, UADT cancer, and prostate cancer mortality in the high compared to the low adherence group. Based on the global Moran’s *I* statistic, only the regression model for liver cancer mortality revealed evidence for spatial autocorrelation. However, the structured spatial component of the INLA model did not indicate evidence for any of the Swiss districts to have a significantly increased or decreased liver cancer mortality.

Our study observed lower all-cause mortality when comparing high to low adherence to the WCRF/AICR score, but did not find an inverse association on the continuous scale as compared to the systematic review by Solans et al. (per 1-point increment: RR = 0.90; 95% CI: 0.84, 0.96; *n* = 3) (32).

In the analyses of all-cancer mortality, we observed that the high compared to the low adherence group had a lower all-cancer mortality. The findings of the systematic review by Solans et al. on the latter association pointed towards the same direction (per 1-point increment: RR = 0.91; 95% CI: 0.89, 0.92; *n* = 3) (32). In addition, our results were in line with those stated in the Swiss study by Lohse et al. (9), who reported a significant decrease in all-cancer mortality by 7% for each 1-point increment in the score (HR = 0.93; 95% CI: 0.90, 0.95) (9). However, the latter results are not directly comparable to ours, since Lohse et al. used an older version of the WCRF/AICR cancer prevention recommendations, included nine instead of seven recommendations, and used more crudely assessed Swiss data on diet and lifestyle (9).

The results of our study regarding colorectal cancer mortality were in contrast to the findings of Romaguera et al. (33), who reported a decrease in colorectal cancer mortality for the high adherence (men: 4–6 points; women: 5–7 points) compared to the low adherence group (men: 0–2 points; women: 0–3 points). Surprisingly, in our study, a borderline significant increase in colorectal cancer mortality was observed for the moderate compared to the low adherence group. The previous Swiss study by Lohse et al. (9) did not find significant evidence for an inverse association.

On the other hand, our study found evidence for a lower mortality from UADT and prostate cancer in the high compared to the low adherence group. These results are in line with the findings of Lohse et al. (9), who reported a decrease in UADT cancer mortality by 51% (HR = 0.49; 95% CI: 0.26, 0.92) and in prostate cancer mortality by 52% (HR = 0.48; 95% CI: 0.28, 0.82) when comparing high (5–9 points) with low adherence (0–3 points). In contrast, there was no association between adherence to WCRF/AICR recommendations and prostate cancer incidence in the systematic review by Solans et al. (32). It might be that a healthy lifestyle is more strongly associated with aggressive disease and, hence, prostate cancer mortality than prostate cancer incidence (34).

Moreover, Lohse et al. observed in the high (5–9 points) compared to the low adherence group (0–3 points) a decrease in stomach cancer mortality by 66% (HR = 0.34; 95% CI: 0.14, 0.83) (9). However, our study did not detect such an association.

As the study by Lohse et al. (9), our study did not find evidence for an inverse association of the score with liver, breast, and pancreatic cancer mortality. Nevertheless, an inverse association between adherence to the recommendations and liver and breast cancer risk has been reported in several previous studies (32, 35, 36). Inverse associations with pancreatic cancer mortality have also recently been observed in an analysis of the Prostate, Lung, Colorectal, and Ovarian Cancer Screening Trial (37).

The lack of significance or unexpected associations of the score with mortality in our study could be due to the lack of mortality data for menuCH participants, reverse causation inherent in the cross-sectional study design of the menuCH study, low numbers of observed deaths, and age differences across the adherence groups, leading to less observed deaths in groups with mainly younger participants.

The Swiss mortality data from the years 2015–2019 showed different geographical patterns across causes of death. The underlying causes for these various spatial patterns could be manifold, e.g., differences in diet culture (38), community social capital (39), or socioeconomic factors (39). Based on the local Moran’s *I* statistic and the LISA cluster map, there was significant evidence in four districts to be spatial clusters or spatial outliers. The fixed effects estimates of the INLA model (Table 3) and the estimates of the Quasipoisson regression model for liver cancer mortality (Table 2) were alike. The structured spatial component of the INLA model did not highlight any Swiss district with a significant increase or decrease in liver cancer mortality.

In comparison to the results of the main analysis, the sensitivity analysis excluding the physical activity recommendation resulted in an attenuation of the significance of the investigated associations (Supplementary Table S1). The attenuated results indicate that the physical activity recommendation is an important component of the cancer mortality prevention index, as reported in previous studies (40, 41). However, statistical power might have been reduced in this sensitivity analysis, since the sample size of the high adherence group was reduced by more than half after excluding the physical activity recommendation.

Furthermore, the second sensitivity analysis, including the recommendation on supplement use, lead to an attenuation of the inverse associations, such that they were no longer statistically significant (Supplementary Table S2). However, statistical power might have been reduced, since the sample size of the low adherence group was reduced by more than half in this sensitivity analysis after including the recommendation on supplement use.

Our study had several strengths. The menuCH weighting strategy allowed our final sample of 2057 participants for being representative of a target population of 4,627,878 Swiss residents. The 24HDR and the lifestyle questionnaire provided detailed information in order to operationalize seven of the ten 2018 WCRF/AICR cancer prevention recommendations for our main analyses and to follow the index by Shams-White et al. (3, 25) closely. For instance, we used an adapted NOVA ultra-processed food classification system to assess fast food consumption, used both measured BMI and waist circumference to calculate the healthy weight score, and distinguished the different types of meat to determine the score for red and processed meat consumption.

However, our study had some limitations. First, the menuCH survey is a cross-sectional study, which, by nature, is prone to reverse causation. Given the self-reported dietary data, assessed with two 24HDR, the possibility of recall bias leading to over- and underestimating of dietary intakes cannot be excluded. However, the assessment of consumed foods *via* two non-consecutive 24HDR using the software GloboDiet® has been shown to yield reliable estimates (19, 20). Second, individual mortality data of the menuCH participants was not available. In our analyses we assumed that each menuCH participant was correctly assigned to their district and that the participants were representative for their district’s lifestyle characteristics. Last, even though our analyses were adjusted for several known confounders, residual confounding cannot be ruled out.

To conclude, using the menuCH data our study was able to overcome the main limitations of the Swiss study by Lohse et al. (9), and provide more up-to-date results using the latest version of the WCRF/AICR cancer prevention recommendations and more detailed dietary and lifestyle data. An inverse association of the cancer prevention score with all-cancer, UADT cancer, and prostate cancer mortality was observed, indicating the potential of the recommendations to decrease the burden of cancer in Switzerland. Significant spatial dependencies were detected only for liver cancer mortality. However, based on the structured spatial component of the INLA model, no evidence was seen for any of the Swiss districts to have a significantly increased or decreased liver cancer baseline mortality rate.

## Abbreviations

WCRF, World Cancer Research Fund; AICR, American Institute for Cancer Research; 24HDR, 24-hour dietary recall; FSO, Federal Statistical Office; FSVO, Federal Food Safety and Veterinary Office; IPAQ, International Physical Activity Questionnaire; BMI, Body Mass Index; IARC, International Agency for Research on Cancer; ICD-10, 10th revision of the international classification of diseases; UADT, upper aero-digestive tract; SMR, standardized mortality ratio; MICE, multivariate imputation by chained equations; MC, Monte Carlo; LISA, local indicators of spatial autocorrelation; INLA, Integrated nested Laplace approximation; RR, rate ratio; SD, standard deviation; CI, confidence interval and credible interval, respectively; NA, missing values.
