# Supplementary material for: Is Following a Cancer-Protective Lifestyle Linked to Reduced Cancer Mortality Risk?
Source: Int J Public Health. 2023 Feb 14;68:1605610. doi: 10.3389/ijph.2023.1605610 (PMC9970999; doi:10.3389/ijph.2023.1605610)
Supplement: Supplementary file 1 [file DataSheet1.docx]

**Supplemental Material**

**Title**

Is following a cancer-protective lifestyle linked to reduced cancer mortality risk?

**Journal**

International Journal of Public Health (IJPH)

Table S1: Association of cancer prevention recommendation score^a^ and sex-, age-, and district-specific mortality rate (Switzerland. 2014-2015. n = 2057. unweighted count) (rate ratios and 95% confidence intervals)^b^

|  | WCRF/AICR cancer prevention recommendations score | | | | |  |
| --- | --- | --- | --- | --- | --- | --- |
|  | Continuous |  | Categorical^g^ | | |  |
| Mortality^h^ | Per 1-point increment  RR (95% CI) |  | Low adherence  (ref.) | Moderate adherence  RR (95% CI) | High adherence  RR (95% CI) |  |
| All-cause^c,d^ | 1.00 (0.99, 1.01) |  | 1.00 | 1.00 (0.97, 1.02) | 1.00 (0.96, 1.04) |  |
| All-cancer^c,d^ | 0.99 (0.98, 1.00) |  | 1.00 | 0.99 (0.97, 1.02) | 0.97 (0.93, 1.02) |  |
| UADT cancer^c,d^ | 0.99 (0.96, 1.02) |  | 1.00 | 0.98 (0.92, 1.04) | 1.01 (0.90, 1.12) |  |
| Stomach cancer^c,d^ | 0.97 (0.93, 1.01) |  | 1.00 | 0.96 (0.86, 1.06) | 0.90 (0.73, 1.07) |  |
| Colorectal cancer^c,d^ | 0.99 (0.96, 1.01) |  | 1.00 | 0.99 (0.93, 1.04) | 0.91 (0.82, 1.01) |  |
| Liver cancer^c,d^ | 0.99 (0.95, 1.02) |  | 1.00 | 0.97 (0.90, 1.04) | 1.00 (0.88, 1.12) |  |
| Pancreatic cancer^c,d^ | 1.01 (0.99, 1.04) |  | 1.00 | 1.01 (0.95, 1.06) | 1.04 (0.95, 1.13) |  |
| Breast cancer^c,e^ | 0.98 (0.95, 1.00) |  | 1.00 | 1.02 (0.96, 1.09) | 0.95 (0.86, 1.04) |  |
| Prostate cancer^c,f^ | 1.00 (0.96, 1.04) |  | 1.00 | 0.93 (0.85, 1.01) | 1.08 (0.92, 1.25) |  |

UADT = upper aero-digestive tract; RR = rate ratio; CI = confidence interval; WCRF = World Cancer Research Fund; AICR = American Institute for Cancer Research.

^a^ The cancer prevention recommendation score included the following six recommendations: healthy weight, plant-based diet, limited consumption of fast-food, red and processed meat, sugar sweetened drinks, and alcohol.

^b^ The menuCH participants’ data were weighted as stated in the menuCH weighting strategy [1] for sex, age, major living region in Switzerland, marital status, household size, nationality, weekday, and season of the recall day.

^c^ A Quasipoisson regression model was fitted.

^d^ The analysis included data of both sexes and were further adjusted for sex, age, smoking category, education level, language region, nationality, civil status, and mean energy intake per day in kilocalories.

^e^ The analysis included data of only women and was further adjusted for age, smoking category, education level, language region, nationality, civil status, and mean energy intake per day in kilocalories.

^f^ The analysis included data of only men and was further adjusted for age, smoking category, education level, language region, nationality, civil status, and mean energy intake per day in kilocalories.

^g^ World Cancer Research Fund / American Institute for Cancer Research cancer prevention score categories: low adherence corresponds to a score of 0-<3 points; moderate adherence corresponds to a score of 3-<5 points; high adherence corresponds to a score of 5-7 points.

^h^ From 2015 until 2019, 106,140 all-cause deaths, including 46,220 all-cancer, 4022 colorectal cancer, 2923 UADT cancer, 1525 stomach cancer, 2248 liver cancer, 3841 pancreatic cancer, 3818 breast cancer (among women), and 1913 prostate cancer deaths (among men) were reported.

Table S2: Association of cancer prevention recommendation score^a^ and sex-, age-, and district-specific mortality rate (Switzerland. 2014-2015. n = 2057. unweighted count) (rate ratios and 95% confidence intervals)^b^

|  | WCRF/AICR cancer prevention recommendations score | | | | |  |
| --- | --- | --- | --- | --- | --- | --- |
|  | Continuous |  | Categorical^g^ | | |  |
| Mortality^h^ | Per 1-point increment  RR (95% CI) |  | Low adherence  (ref.) | Moderate adherence  RR (95% CI) | High adherence  RR (95% CI) |  |
| All-cause^c,d^ | 0.99 (0.98, 1.00) |  | 1.00 | 1.04 (1.00, 1.09) | 0.99 (0.94, 1.04) |  |
| All-cancer^c,d^ | 0.99 (0.98, 1.00) |  | 1.00 | 1.05 (1.00, 1.11) | 1.00 (0.95, 1.06) |  |
| UADT cancer^c,d^ | 0.99 (0.97, 1.02) |  | 1.00 | 1.02 (0.90, 1.14) | 0.97 (0.85, 1.09) |  |
| Stomach cancer^c,d^ | 0.98 (0.94, 1.02) |  | 1.00 | 1.10 (0.91, 1.29) | 1.00 (0.81, 1.20) |  |
| Colorectal cancer^c,d^ | 0.98 (0.96, 1.00) |  | 1.00 | 1.03 (0.92, 1.14) | 0.98 (0.87, 1.09) |  |
| Liver cancer^c,d^ | 1.00 (0.97, 1.03) |  | 1.00 | 1.16 (1.02, 1.31) | 1.11 (0.96, 1.26) |  |
| Pancreatic cancer^c,d^ | 1.00 (0.98, 1.03) |  | 1.00 | 1.11 (1.00, 1.22) | 1.09 (0.98, 1.20) |  |
| Breast cancer^c,e^ | 0.98 (0.96, 1.01) |  | 1.00 | 1.20 (1.07, 1.34) | 1.15 (1.02, 1.29) |  |
| Prostate cancer^c,f^ | 0.98 (0.95, 1.01) |  | 1.00 | 0.93 (0.78, 1.07) | 0.82 (0.67, 0.97) |  |

UADT = upper aero-digestive tract; RR = rate ratio; CI = confidence interval; WCRF = World Cancer Research Fund; AICR = American Institute for Cancer Research.

^a^ The cancer prevention recommendation score included the following eight recommendations: healthy weight, physical activity, plant-based diet, limited consumption of fast-food, red and processed meat, sugar sweetened drinks, alcohol, and supplement intake.

^b^ The menuCH participants’ data were weighted as stated in the menuCH weighting strategy [1] for sex, age, major living region in Switzerland, marital status, household size, nationality, weekday, and season of the recall day.

^c^ A Quasipoisson regression model was fitted.

^d^ The analysis included data of both sexes and were further adjusted for sex, age, smoking category, education level, language region, nationality, civil status, and mean energy intake per day in kilocalories.

^e^ The analysis included data of only women and was further adjusted for age, smoking category, education level, language region, nationality, civil status, and mean energy intake per day in kilocalories.

^f^ The analysis included data of only men and was further adjusted for age, smoking category, education level, language region, nationality, civil status, and mean energy intake per day in kilocalories.

^g^ World Cancer Research Fund / American Institute for Cancer Research cancer prevention score categories: low adherence corresponds to a score of 0-<3 points; moderate adherence corresponds to a score of 3-<5 points; high adherence corresponds to a score of 5-7 points.

^h^ From 2015 until 2019, 106,140 all-cause deaths, including 46,220 all-cancer, 4022 colorectal cancer, 2923 UADT cancer, 1525 stomach cancer, 2248 liver cancer, 3841 pancreatic cancer, 3818 breast cancer (among women), and 1913 prostate cancer deaths (among men) were reported.

Table S3: Global Moran's *I* statistic based on Quasipoisson regression model residuals at the district level (Switzerland. 2014-2015 and 2015-2019, respectively. n = 75) using the continuous score^a^

| Cause of death^c^ | Observed Moran’s *I* | Expected Moran’s *I* | Variance Moran’s *I* | *P*_Z-score_^b^ | *P*_MC_^b^ |
| --- | --- | --- | --- | --- | --- |
| All-cause | 0.026 | -0.014 | 0.007 | 0.32 | 0.30 |
| All-cancer | 0.051 | -0.014 | 0.007 | 0.22 | 0.22 |
| UADT cancer | 0.01 | -0.014 | 0.006 | 0.38 | 0.38 |
| Stomach cancer | 0.086 | -0.014 | 0.006 | 0.10 | 0.10 |
| Colorectal cancer | -0.035 | -0.014 | 0.007 | 0.40 | 0.43 |
| Liver cancer | 0.152 | -0.014 | 0.006 | 0.017 | 0.027 |
| Pancreatic cancer | -0.117 | -0.014 | 0.007 | 0.11 | 0.11 |
| Breast cancer | -0.129 | -0.014 | 0.008 | 0.097 | 0.086 |
| Prostate cancer | 0.057 | -0.014 | 0.008 | 0.21 | 0.19 |

UADT = upper aero-digestive tract, MC = Monte Carlo; WCRF = World Cancer Research Fund; AICR = American Institute for Cancer Research.

^a^ The cancer prevention recommendation score included the following seven recommendations: healthy weight, physical activity, plant-based diet, limited consumption of fast-food, red and processed meat, sugar sweetened drinks, and alcohol.

^b^ One-sided *P* value with significance level $\alpha$= 0.05.

^c^ From 2015 until 2019, 106,140 all-cause deaths, including 46,220 all-cancer, 4022 colorectal cancer, 2923 UADT cancer, 1525 stomach cancer, 2248 liver cancer, 3841 pancreatic cancer, 3818 breast cancer (among women), and 1913 prostate cancer deaths (among men) were reported.

Table S4: Global Moran's *I* statistic based on Quasipoisson regression model residuals aggregated at the district-level (Switzerland. 2014-2015 and 2015-2019, respectively. n = 75) using the categories of adherence to the score^a^

| Cause of death^c^ | Observed Moran’s *I* | Expected Moran’s *I* | Variance Moran’s *I* | *P*_Z-score_^b^ | *P*_MC_^b^ |
| --- | --- | --- | --- | --- | --- |
| All-cause | 0.032 | -0.014 | 0.007 | 0.30 | 0.28 |
| All-cancer | 0.049 | -0.014 | 0.007 | 0.23 | 0.23 |
| UADT cancer | 0.006 | -0.014 | 0.006 | 0.40 | 0.40 |
| Stomach cancer | 0.086 | -0.014 | 0.006 | 0.10 | 0.098 |
| Colorectal cancer | -0.042 | -0.014 | 0.007 | 0.37 | 0.40 |
| Liver cancer | 0.143 | -0.014 | 0.006 | 0.023 | 0.033 |
| Pancreatic cancer | -0.115 | -0.014 | 0.007 | 0.11 | 0.11 |
| Breast cancer | -0.13 | -0.014 | 0.008 | 0.096 | 0.088 |
| Prostate cancer | 0.059 | -0.014 | 0.008 | 0.21 | 0.19 |

UADT = upper aero-digestive tract, MC = Monte Carlo; WCRF = World Cancer Research Fund; AICR = American Institute for Cancer Research.

^a^ The cancer prevention recommendation score included the following seven recommendations: healthy weight, physical activity, plant-based diet, limited consumption of fast-food, red and processed meat, sugar sweetened drinks, and alcohol. World Cancer Research Fund / American Institute for Cancer Research cancer prevention score categories: category 1: low adherence (0-<3 points); category 2: moderate adherence (3-<5 points); category 3: high adherence (5-7 points).

^b^ One-sided *P* value with significance level $\alpha$= 0.05.

^c^ From 2015 until 2019, 106,140 all-cause deaths, including 46,220 all-cancer, 4022 colorectal cancer, 2923 UADT cancer, 1525 stomach cancer, 2248 liver cancer, 3841 pancreatic cancer, 3818 breast cancer (among women), and 1913 prostate cancer deaths (among men) were reported.

**References**

[1] Pasquier J, Chatelan A, Bochud M. Weighting strategy (2017). https://menuch.unisante.ch/index.php/catalog/4/download/17 [Accessed November 18, 2022].
